# Genotoxic Evaluation of Fe_3_O_4_ Nanoparticles in Different Three Barley (*Hordeum vulgare* L.) Genotypes to Explore the Stress-Resistant Molecules

**DOI:** 10.3390/molecules26216710

**Published:** 2021-11-05

**Authors:** Inese Kokina, Ilona Plaksenkova, Renata Galek, Marija Jermaļonoka, Elena Kirilova, Vjaceslavs Gerbreders, Marina Krasovska, Eriks Sledevskis

**Affiliations:** 1 Department of Biotechnology, Institute of Life Sciences and Technology, Daugavpils University, Parades Str. 1A, LV-5401 Daugavpils, Latvia; inese.kokina@du.lv (I.K.); marija.jermalonoka@du.lv (M.J.); 2 Department of Genetics, Plant Breeding and Seed Science, Wroclaw University of Environmental and Life Sciences, Grunwaldzki Sq. 24A, 50-363 Wroclaw, Poland; renata.galek@upwr.edu.pl; 3 Department of Applied Chemistry, Institute of Life Sciences and Technology, Daugavpils University, Parades Str. 1A, LV-5401 Daugavpils, Latvia; jelena.kirilova@du.lv; 4 Department of Technology, Institute of Life Sciences and Technology, Daugavpils University, Parades Str. 1A, LV-5401 Daugavpils, Latvia; vjaceslavs.gerbreders@du.lv (V.G.); marina.krasovska@du.lv (M.K.); eriks.sledevskis@du.lv (E.S.)

**Keywords:** miRNAs, nanonutrition, sustainable agriculture, plant nanobiotechnology

## Abstract

Sustainable agricultural practices are still essential due to soil degradation and crop losses. Recently, the relationship between plants and nanoparticles (NPs) attracted scientists’ attention, especially for applications in agricultural production as nanonutrition. Therefore, the present research was carried out to investigate the effect of Fe_3_O_4_ NPs at low concentrations (0, 1, 10, and 20 mg/L) on three genotypes of barley (*Hordeum vulgare* L.) seedlings grown in hydroponic conditions. Significant increases in seedling growth, enhanced chlorophyll quality and quantity, and two miRNA expression levels were observed. Additionally, increased genotoxicity was observed in seedlings grown with NPs. Generally, Fe_3_O_4_ NPs at low concentrations could be successfully used as nanonutrition for increasing barley photosynthetic efficiency with consequently enhanced yield. These results are important for a better understanding of the potential impact of Fe_3_O_4_ NPs at low concentrations in agricultural crops and the potential of these NPs as nanonutrition for barley growth and yield enhancement. Future studies are needed to investigate the effect of these NPs on the expression of resistance-related genes and chlorophyll synthesis-related gene expression in treated barley seedlings.

## 1. Introduction

According to a Food and Agriculture Organisation (FAO) report, many people still experience food shortages. Moreover, the requirements for food will gradually increase in the world, at least until 2030 [1]. Permanent pesticide, herbicide, and fungicide use, and climate change induce soil degradation and crop losses [2,3]. Therefore, sustainable agricultural practices are essential for humankind [3]. Recently, the relationship between plants and nanoparticles (NPs) has attracted scientists’ attention, especially to its application in agricultural production as nanonutrition. It has been shown that a small number of different NPs are present in agricultural soil which have been shown to accumulate in plants [1].

Research has explored that NPs, due to their small size (<100 nm), can pass plant biological barriers, thus allowing efficient delivery of nutrients/micronutrients [4]. Therefore, NPs, as a versatile resource of plant nanobiotechnology, can improve the management of crop disease and crop loss, thereby increasing plant resistance to various environmental biotic and abiotic stressors. Moreover, this method reduces the use of chemical fertilisers and soil contamination and degradation, promoting sustainable agricultural production [3,4,5,6,7,8].

Micronutrients are essential to protect crops from plant pathogens and the diseases they cause [9,10]. Iron (Fe) is an essential nutrient used for chlorophyll synthesis, photosynthesis, and respiration [11]. Nevertheless, the uptake of iron oxide (Fe_3_O_4_) NPs is limited in plants due to the low solubility of these NPs in water. Fe-based NPs can increase photosynthesis and chlorophyll in several plants, such as maize, soybeans, rice, barley, and yellow medick [1,12,13]. Moreover, Fe-based NPs increase plant germination and growth [10,14]. Iron deficiency affects crop quality and production [15]. Fe_3_O_4_ nanoparticles have many special properties, such as low toxicity, superparamagnetism, and biodegradability [16].

Plants are often exposed to many biotic (pathogens, herbivore) and abiotic (extreme temperatures, salinity, drought) stressors that reduce average yields and crop quality by about 30% per year [17] or about 50% worldwide [3,10,18]. Plants have developed complex systems of protection against infections that are activated by recognising pathogen-associated molecular models (PAMPs) or pathogen effectors that produce PAMP-triggered immunity (PTI) and effector-triggered immunity (ETI). With the help of PTI and ETI, plants can protect themselves against viral, fungal, oomycete, and bacterial infections [17]. The pathogen releases specific proteins upon entry into the host cell, and the host induces a mechanism of resistance as a response. One of the mechanisms of PTI is the silencing mechanism of RNA, which is used in plant protection [19].

Plants have specific microRNA (miRNA) sequences that are involved in regulating gene expression and protecting cells against invasive nucleic acids. miRNAs are small (<24 nucleotides) non-coding RNA molecules that bind to target mRNAs, thus inhibiting post-transcriptional translation. miRNAs are engaged in the regulation of many biological processes in eukaryotic organisms, such as cell proliferation, apoptosis, differentiation, and gene expression [20,21]. Furthermore, miRNAs are involved in the response to both abiotic and biotic stressors to provide immunity to pathogens. During stress, changes in both gene and miRNA expression levels appear, promoting defense responses and resistance in plants [12,22,23]. According to reports, transcription factors are the targets of miR159 that control plant development, morphology, and flowering [24]. Furthermore, miR159c is known to be involved in the response to fungal infection in wheat [25]. However, miR156 can be involved in resistance to viral infection [26]. According to Yao et al. (2021) [27], miR156 and miR159 families’ targets are at least three genes that are related to the resistance of Tibetan hulless barley against fungal diseases, such as barley leaf stripe [27]. In addition, different expression patterns of miR156 and miR159 have been detected in barley exposed to abiotic stress, such as drought, salt, and dehydration stress [27,28].

Cereals are known to be the most relevant food component since they have a high carbohydrate content, provide vitamins, trace minerals, dietary fibre, proteins, antioxidants, and bioactive compounds [29]. Barley (*Hordeum vulgare* L.) is an annual crop plant, which is among the oldest cultivated crops in the world, grown in the semi-arid subtropics and temperate climates [30]. Presently, barley has great agronomical importance and high economic value. It ranks fourth in both quantities produced and in the area of cultivation of cereal crops in the world. Barley is used in food and industry, such as in beer production, malting, and animal feed [29,31,32]. Unfortunately, there is a serious risk of disease in barley, such as powdery mildew caused by *Blumeria graminis*. This disease is one of the most destructive barley diseases in the world and results in yield losses of up to 50% [33,34]. Therefore, in this study, three different barley varieties with different resistance genes, namely *mlo*, *mla*, and without resistance genes, were evaluated.

Several studies have been conducted to explore the effects of various NPs on different crop properties with the view of crop disease suppression and yield enhancement. However, the ability of some NPs to enhance crop protection is limited. The involvement of miRNAs, such as miR156 and miR159, in the plant response to NPs is still limited [35]. Therefore, the aim of the present research was to clarify the potential of Fe_3_O_4_ nanoparticles as an effective tool to improve growth and increase the chlorophyll content and expression of resistance-related molecules against biotic and abiotic stressors in different barley genotypes.

## 2. Materials and Methods

### 2.1. Preparation of Fe_3_O_4_ Nanoparticles and Their Characteristics

Fe_3_O_4_ NPs with an average size of 25 nm were provided by G. Libert’s Center of Innovative Microscopy, Daugavpils University. Fe_3_O_4_ nanoparticles were obtained by the co-precipitation (Massart) method [36] using ferric chloride (II) and (III) in a ratio of at least 1:2 and aqueous ammonium hydroxide solution. For this purpose, 0.167 g of FeCl_3_·6H_2_O and 0.0429 g of FeCl_2_·4H_2_O were dissolved in 50 mL of distilled water. 0.27 mL of 25% NH_4_OH was added dropwise to the solution under constant stirring. The obtained nanostructures were stabilized with an aqueous citric acid solution (40 mg/mL, 2 mL). The resulting precipitate was separated with a permanent magnet and rinsed three times with distilled water to remove residual reagents. Schematically, the production of Fe_3_O_4_ nanostructures can be described by the following equation:

Fe^2+^ + 2Fe^3+^ + 8OH^−^ = Fe_3_O_4_↓ + 4H_2_O

**Figure 1:**
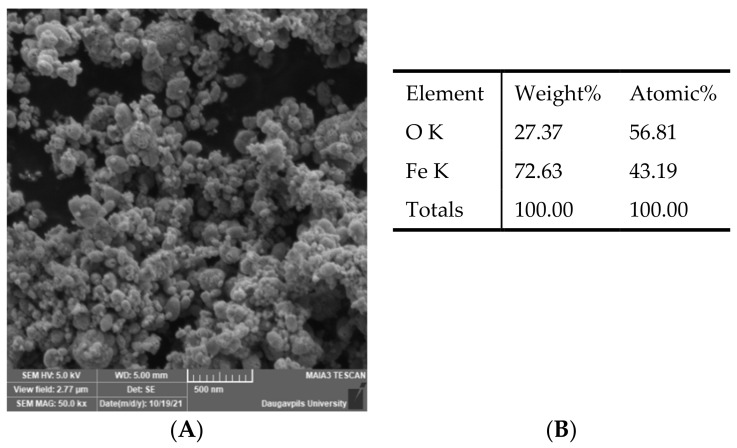
SEM image and chemical composition of Fe_3_O_4_ nanoparticles. (**A**)—SEM image of Fe_3_O_4_ nanoparticles; (**B**)—chemical composition of Fe_3_O_4_ nanoparticles.

The morphology of the Fe_3_O_4_ NPs was studied by the Field Emission Scanning Electron Microscopy (SEM) (MAIA 3, Tescan, Czech Republic). The chemical composition of the nanoparticles was researched by EDS installation (Inca, Oxford Instruments, UK). It can be seen from the SEM image (Figure 1) that the Fe_3_O_4_ powder consists of agglomerates of individual nanoparticles.

The crystalline structure of the samples was defined by an X-ray diffractometer (Smart Lab, Rigaku, Japan) and corresponds to magnetite (Fe_3_O_4_). The size of individual nanoparticles was determined using PDXL software according to the Williamson–Hall method and its value was 25 nm.

### 2.2. Barley Seedling Cultivation

Latvian origin spring barley genotypes ‘Sencis’ *(mla 13)*, ‘Abava’ (lack of resistance genes), and commonly used high yielding spring malting England variety ‘Quench’ *(mlo 11)* were grown for the research. The seeds were provided by the Institute of Agricultural Resources and Economics, Stende Research Center (Priekuli, Latvia). The seeds were rinsed with deionized water and transferred for germination to a hydroponic tray for 8 days at 22 °C. The seedlings were transferred to tubes with tap water (control) supplemented with different Fe_3_O_4_ NPs solutions (experimental). An equivalent number of plants (n = 30) were used in each treatment (control and three experimental groups) and each stage of the experiment (for morphological parameters, chlorophyll, genotoxicity, and miRNA analysis). Barley seedlings were grown in NP solution conditions for 8 days.

Nanoparticles were diluted in water to 1, 10, and 20 mg/L and sonicated for 30 min to split the formed nanoparticle agglomerates into individual NPs before use in the experiment.

### 2.3. Detection of Fe_3_O_4_ Nanoparticles in Barley Using Confocal Laser-Scanning Fluorescence Microscopy (CLSM)

Fluorescent Fe_3_O_4_ NPs (25 nm) were purchased from Sigma-Aldrich (Germany). The experiment with fluorescent Fe_3_O_4_ NPs was prepared as described in Section 2.1. CLSFM was performed with a Nikon Eclipse Ti-E microscope (Nikon, Tokyo, Japan). Fluorescence was excited at *λ* = 457.9 nm, and emission was detected at 464–499 nm. Confocal images were obtained with NIS-elements Advanced Research 3.2 64-bit software (Nikon). Parameters for magnification at 200× and 600× were the same for all images and were as follows: HV = 154, offset = −34, laser power = 63.4. Detection of NPs was performed on day 1 and 6.

### 2.4. Effect of Fe_3_O_4_ Nanoparticles on Barley Morphological Parameters

The shoot length and root length of barley seedlings were measured, and the number of roots was counted after 8 days of growth in water (control) with the application of different concentrations of Fe_3_O_4_ NPs (1, 10, and 20 mg/L). The mean length values were calculated using three biological replicates.

### 2.5. Effect of Fe_3_O_4_ Nanoparticles on Chlorophyll Content and the Steady State Absorption and Fluorescence Spectra of PSII and PSI in Treated Barley Seedlings

Chlorophyll extraction from control and treated barley seedlings’ leaves was performed to determine chlorophyll content and to estimate the fluorescence spectra of PSII and PSI. Leaves (300 mg fresh weight) from each group were cut into pieces, ground with a mortar and pestle, and mixed with 5 mL 96% ethanol. Thereafter, the mixture was incubated in the dark for 30 min, followed by centrifugation for 10 min at 3000 rpm. The collected supernatant was kept in the dark until sample analysis. Three replicates for each treatment were performed.

Samples were analysed with a UV-Visible two-beam spectrophotometer SHIMADZU UV-2550PC (Shimadzu Corporation, Kioto, Japan). The absorbance of each treatment sample was recorded at 645 and 663 nm wavelengths for chlorophyll *a* and chlorophyll *b*, respectively. The measurement error was less than 2%. Chlorophyll content was estimated by Arnon’s Equations (1) and (2) [37,38]:

(1) $$\mathrm{Chl}\;a\;\left(\mathrm{mg}/g\right)=\left[12.7\times A663-2.69\times A645\right]\times \frac{V}{1000}\times W$$

(2) $$\mathrm{Chl}\;b\;\left(\mathrm{mg}/g\right)=\left[22.9\times A645-4.86\times A663\right]\times \frac{V}{1000}\times W$$

where V—volume of extract in mL; W—fresh weight of leaves in mg; A663—solution absorbance at 663 nm; A645—solution absorbance at 645 nm.

Steady-state fluorescence measurements of the samples were performed in ethanol solutions, detecting the photoluminescence signal in a 90° configuration with a spectrofluorometer from Edinburgh Instruments Ltd. (Livingston, UK), model FLSP920 in the range 600–800 nm (excitation wavelength at 440 nm). The spectroscopic measurements were performed at an ambient temperature, and the solutions were placed in a quartz cuvette with a 1 cm path length and four polished windows. Fluorescence spectra were detected at 675 nm for PSII and at 730 nm for PSI.

### 2.6. Effect of Fe_3_O_4_ Nanoparticles on Barley Genome DNA Using the Comet Assay

A comet assay was conducted according to Trevigen’s CometAssay^®^ (Trevigen, Gaithersburg, MD, USA) protocol for an alkaline comet assay with slight modifications. Fresh tissues of roots and leaves were minced with scissors into very small pieces and collected in Eppendorf tubes containing ice-cold PBS. The cell suspensions were combined with molten low melting agarose (LMAgarose) according to the manufacturer’s protocol. After electrophoresis, slides were immersed twice in dH_2_O for 5 min each, then in 70% ethanol for 5 min, and dried at 37 °C. Nucleoids were stained with SYBR^®^ Green and analyzed with a fluorescence microscope (Nikon Eclipse 80i, Tokyo, Japan) using filters at 465–495 nm (excitation). The comet “tails” were detected and analysed using the LUCIA Comet Assay^TM^ (LUCIA, Praha, Czech Republic) software, scoring 300 comets for each treatment group under a 40× magnification.

### 2.7. Effect of Fe_3_O_4_ Nanoparticles on Barley miRNA Expression Using qRT-PCR

The total RNA was isolated and purified from fresh, treated barley seedling leaves and roots using a Universal RNA/miRNA Purification Kit (EURx, Gdańsk, Poland) according to the manufacturer’s protocol. The RNA concentration and quality were determined using a NanoDrop One spectrophotometer (Thermo Fisher Scientific, Waltham, MA, USA) at OD 260/280 and OD 260/230 absorbance ratios. The first-strand cDNA was synthesized from 1 μg of total RNA using a miRCURY LNA RT Kit (Qiagen, Hilden, Germany) according to the manufacturer’s instructions.

The quantitative real-time RT-PCR (qRT-PCR) method was used to evaluate the expression of miRNAs in treated barley seedlings. qRT-PCR was performed on the Rotor-Gene Q (Qiagen, Hilden, Germany). miRCURY SYBR Green PCR reagents (Qiagen, Hilden, Germany) were utilized to perform a miRNA qRT-PCR analysis according to the manufacturer’s instructions. UniSp6 RNA was used as an internal control. MicroRNA target-specific primers lus-miR159c, hvu-miR159a, and hvu-miR156a with locked nucleic acids were purchased. miRNA target sequences were as follows: lus-miR159c: 5′-UUUGGAUUGAAGGGAGCUCUU-3′; hvu-miR159a: 5′-UUUGGAUUGAAGGGAGCUCUG-3′; and hvu-miR156a: 5′-UGACAGAAGAGAGUGAGCACA-3′.

Barley *HvsnoR14* was used as a reference gene for data normalization. The relative expression of miRNAs in different samples compared to that of the controls was calculated using the 2−ΔΔCt method [39], for which the Ct value was the average of three biological replicates with three technical replicates.

### 2.8. Statistical Analyses

The results were expressed as the mean of the measurements and presented as the mean ± standard deviation (SD). A two-way analysis of variance (ANOVA) was conducted to determine the significance of barley genotype, doses of Fe_3_O_4_ nanoparticles, and their interaction on barley seedling’s reaction in regard to morphological parameters, chlorophyll content, and genotoxicity based on a comet assay.

The hypothesis presumes no impact of genotype, dose of Fe_3_O_4_ nanoparticles, or interactions on the estimated parameters. The significant differences were assessed at a *p*-value of 0.05 and 0.01. When ANOVA gave a significant result, Tukey’s HSD test was performed at the 0.05 level to compare the mean values in cases where the hypothesis was rejected [40]. The obtained results were subject to statistical analysis using the Statistica program, version 13.3.

## 3. Results and Discussion

### 3.1. Fe_3_O_4_ Nanoparticle Translocation in Barley Seedlings

**Figure 2:**
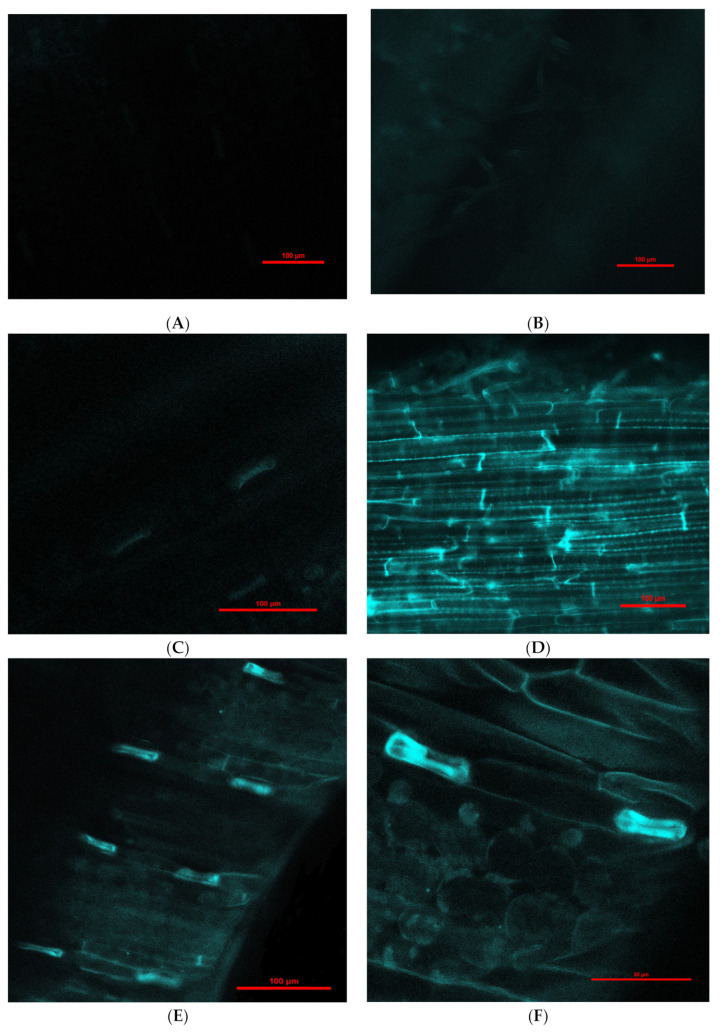

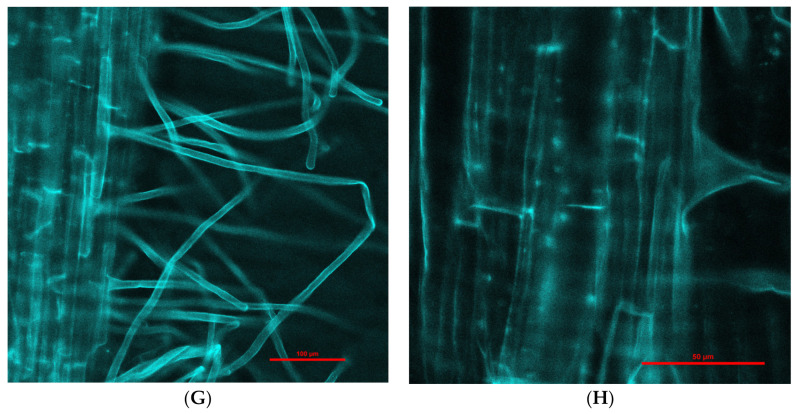
Examples of confocal laser scanning images of barley seedling leaves (**A**,**C**,**E**,**F**) and roots (**B**,**D**,**G**,**H**) treated hydroponically with Fe_3_O_4_ NPs. (**A**)—control seedling leaves (magnification 200×); (**B**)—control seedling root (magnification 200×); (**C**)—presence of fluorescent Fe_3_O_4_ NPs in seedling leaves after 1-day treatment (magnification 200×); (**D**)—presence of fluorescent Fe_3_O_4_ NPs in seedling roots after 1-day treatment (magnification 200×); (**E**)—presence of fluorescent Fe_3_O_4_ NPs in seedling leaves after 6-day treatment (magnification 200×); (**F**)—presence of fluorescent Fe_3_O_4_ NPs in seedling leaves after 6-day treatment (magnification 600×); (**G**)—presence of fluorescent Fe_3_O_4_ NPs in seedling roots after 6-day treatment (magnification 200×); (**H**)—presence of fluorescent Fe_3_O_4_ NPs aggregates (rows) in seedling roots after 6-day treatment (magnification 600×).

Engineered nanoparticles can penetrate plant root cells by different mechanisms, such as through aquaporins, membrane transport proteins, endocytosis, or creating new pores [41,42]. Our experiment with purchased fluorescent Fe_3_O_4_ NPs that were 25 nm in diameter (this was the smallest size available for purchase due to the pandemic) showed penetration into barley roots and translocation to leaves (Figure 2). Moreover, there was a difference in fluorescence intensity between leaves exposed to NPs for 1 (Figure 2C,D) and 6 days (Figure 2E–H); longer treatment with NPs showed greater fluorescence in leaves in comparison to 1-day treatment. Fluorescence in control roots and leaves was not detected.

The cell wall and plasma membrane are selective barriers that control the entry of NPs into plant cells. Therefore, the penetration of NPs into plant cells depends on the composition and size of the NP, plant species, diameter of cell wall pores, and endocytic vesicles [42,43,44,45]. Moreover, there is a possibility that different plant species have different cell pore sizes [43,45]. For example, in cotton seedlings, NPs up to 20 nm may enter leaf cells; however, for maize seedlings, NPs entry is limited to 11 nm [45]. Ma et al. (2010) [46] suggested that 20 nm AgNPs from intercellular spaces can be transported into plant cells through plasmodesmata. Tombuloglu et al. (2019) [47] confirmed the uptake and translocation of 13 nm Fe_3_O_4_ NPs in barley seedlings. Palocci et al. (2017) [42] identified NP size limits to enter the cell wall and plasma membrane in grapevine cell cultures. Researchers have shown that poly(lactic-co-glycolic) acid NPs with diameters up to 50 and 540 nm are able to cross the cell wall and plasma membrane, respectively. Further analysis confirmed that these NPs are taken up by endocytic vesicles [42]. Investigations performed by Bandmann and Homann (2012) [48] revealed that the diameter of tobacco protoplast vesicles was between 80 and 220 nm. It seems that different species have different sizes of endocytic vesicles [42]. Interestingly, researchers have also presumed that NPs interact with the cell wall and induce the formation of new and large pores that allow penetration into plant cells [46]. Wang et al. (2012) [49] suggested that the pore sizes of plant walls are only 3–8 nm.

Our experiment with purchased fluorescent Fe_3_O_4_ NPs that were 25 nm in diameter (this was the smallest size available for purchase due to the COVID-19 pandemic) showed penetration into barley roots and translocation to leaves (Figure 2). Moreover, there was a difference in fluorescence intensity between leaves exposed to NPs for 1 and 6 days; longer treatment with NPs showed greater fluorescence in leaves in comparison with 1-day treatment. Fluorescence in control roots and leaves was not detected. According to some scientists, it is possible that 25 nm NPs are able to penetrate plant cells [42,48]. Experiments with 52 nm Fe NPs demonstrated that these NPs had not been transported into red pepper leaves by the vascular tissues. In contrast, these NPs penetrated the extracellular space of red pepper plants and reached the endodermis, which indicates these NPs can move through the apoplastic pathway in the roots [50]. Furthermore, agglomerated Fe_2_O_3_ and Fe_3_O_4_ NPs can migrate to the endodermis and accumulate in the vacuole in corn via the apoplastic pathway [51,52]. In red pepper plants, Fe NPs can be modified into iron ions and transported through the vascular tissues to leaves [50]. In our case, we could not demonstrate the penetration of Fe_3_O_4_ NPs (25 nm) into plant cells, but it seems that these NPs were transported to barley leaves through the apoplastic pathway.

### 3.2. Effect of Fe_3_O_4_ Nanoparticles on Barley Seedling Growth

**Figure 3:**
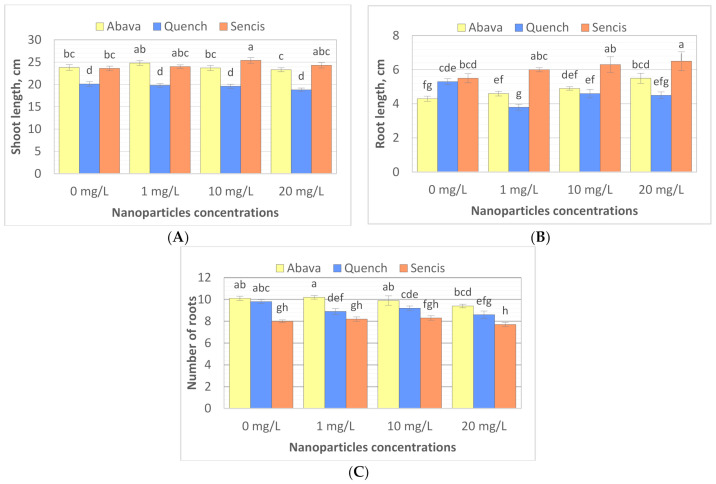
Shoot length (**A**), root length (**B**), number of seminal roots (**C**) expressed as the % of control, in *H. vulgare* L. cultivars seedlings grown 8 days with 1, 10, and 20 mg/L of Fe_3_O_4_ NPs. Values are the mean of three replicates with SD. Different letters within each bar indicate significant differences at *p* < 0.05 and the same letters indicate no significant difference (Tukey’s test—two-way analysis of variance).

To study the effect of Fe_3_O_4_ NPs on barley seedling growth, the shoot and root lengths of seedlings were measured, and the number of roots was counted after 8 days of exposure to Fe_3_O_4_ NPs (25 nm). The results showed that different Fe_3_O_4_ NP concentrations significantly influenced plant morphology of the three tested barley cultivars (Figure 3A–C) compared to control samples. Additionally, significant differences were observed between plant shoot length, root length, and root number in control samples (Figure 3A–C). The shoot length of non-treated plants varied from 20.1 (‘Quench’) to 23.8 cm (‘Abava’). ‘Abava’ was characterised by 1 cm shorter roots (4.3 cm) than the other two cultivars, but they were more numerous (10.1). NP concentrations generally significantly increased the shoot (23.8 to 24.8 and 23.6 to 25.4 cm) and root length (4.3 to 5.5cm and 5.5 to 6.5 cm) of ‘Abava’ and ‘Sencis’, respectively. Treated ‘Quench’ plants had lower parameters of growth compared to the control, with reductions of1.3, 0.8, and 1.2 cm, depending on the trait (Figure 3A–C). Treated ‘Sencis’ plants had the highest growth dynamics, especially at 10 mg/L (Figure 3A–C). ‘Abava’ seedlings had a 1 cm greater increase in shoot length and a 0.1 increase in the number of roots at the lowest dose. Increasing the nanoparticle dose, especially to 20 mg/L, resulted in significant root growth (up to 1.2 cm in ‘Abava’seedlings).

Our previous study on two different barley genotypes showed a significant increase in seedling growth when they were treated with 17 mg/L Fe_3_.O_4_ NPs [53]. Konate et al. (2017) [54] observed the slight enhancement of root and shoot length in wheat seedlings exposed to small (7 nm) Fe_3_O_4_ NPs compared to the control. Wang et al. (2019) [55] found that initially, 400 mg/L Fe_3_O_4_ NPs had an inhibitory effect on muskmelon growth, but after the third week of treatment, growth promotion was observed. According to Rahmatizadeh et al. (2019) [56], enhanced growth of tomato plants was observed with up to 100 mg/L Fe_3_O_4_ NPs compared to the control after 2 weeks of exposure. Yan et al. (2020) [57] made similar observations on maize; in the experiment, a significant increase in plant root length was observed after treatment with doses of Fe_3_O_4_ NPs, such as 50 and 500 mg/kg of soil, for 4 weeks. Additionally, Pariona et al. (2017) [52] claimed that Fe_3_O_4_ NPs increased the growth of oak plants for up to 12 weeks. Tombuloglu et al. (2019) [47] revealed that Fe_3_O_4_ NPs at concentrations up to 250 mg/L enhanced the growth of barley seedlings after 3 weeks of treatment. Leaf length was increased by 27%, and root length increased 125% compared to control seedlings. Yellow medick seedlings exposed to the same NPs at 4 mg/L for 5 weeks showed significantly increased root length and number of leaves [12]. Even low Fe_3_O_4_ NPs concentrations (1, 2, and 4 mg/L) significantly increased shoot and root length in garden rockets exposed to NPs for 5 weeks [58]. Yuan et al. (2018) [50] reported that Fe NPs (52 nm) promoted red pepper plant height at lower concentrations (0.05 mM/L). Furthermore, Trujillo-Reues et al. (2014) [59] showed no effect of Fe_3_O_4_ NPs at 10 and 20 mg/L on lettuce growth after 15 days of exposure. In contrast, Wang et al. (2012) [49] showed that Fe_3_O_4_ NPs (20 nm) at 1000 and 2000 mg/L did not have a significant effect on root elongation in lettuce seedlings on the fourth day of exposure. Moreover, Ghafariyan et al. (2013) [60] showed a significant decrease in soybean root elongation after treatment with iron oxide NPs at concentrations above 200 mg/L.

Plant growth reduction could be related to Fe NP aggregation on the surface of roots, which interrupts water uptake [59]. Phytotoxicity can occur if NPs penetrate cell walls and plasma membranes in plant roots and then enter vascular tissues [49]. Fe_3_O_4_ is affected by oxidation in the presence of water and oxygen, which results in the accumulation of OH radicals and cell wall loosening followed by cell elongation. There can be limited NP transfer to leaves of seedlings [47]. Our results clearly indicated that Fe_3_O_4_ NPs had a significant impact on plant height compared with the control.

### 3.3. Content of Chlorophyll in Barley Seedlings after Treatment with Fe_3_O_4_ Nanoparticles

**Figure 4:**
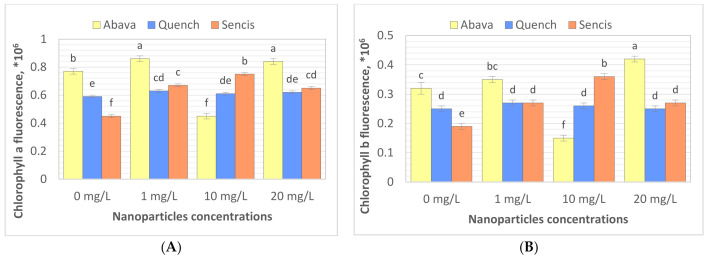

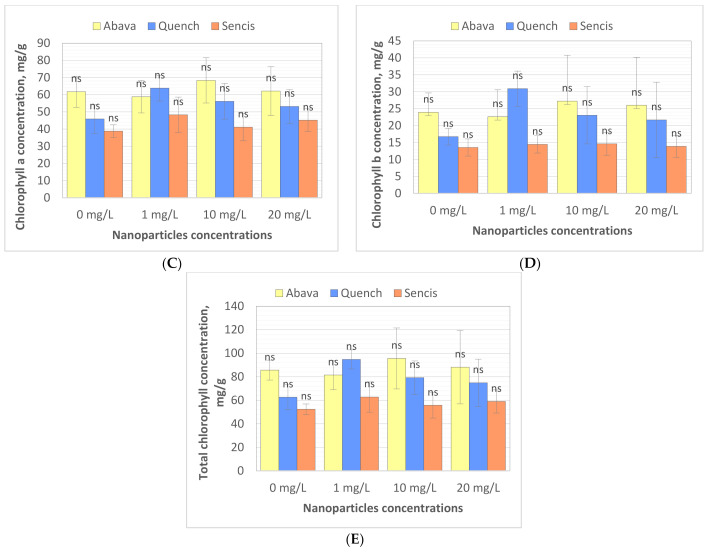
Effects of different Fe_3_O_4_ nanoparticle concentrations on chlorophyll a, and b fluorescence (×106) (**A**,**B**), content (mg/g) (**C**,**D**), and total chlorophyll concentration (mg/g) (**E**) in treated three *H. vulgare* L. seedlings expressed as the % of control. Values are the mean of three replicates with SD. Different letters within each bar indicate significant differences at *p* < 0.05 and the same letters or ‘ns’ indicate no significant difference (Tukey’s test—two-way analysis of variance).

Chlorophyll (Chl) is an important photosynthetic pigment in plants that participates in photosynthesis and thus plant growth [61]. Chl content is a key parameter for many plant biological studies [62]. To identify the effects of the Fe_3_O_4_ NPs on the barley seedling Chl level, Chl quality (fluorescence, ×10^6^) and quantity (content of chlorophyll, mg/g) were determined. All tested concentrations significantly affected Chl fluorescence (Figure 4A,B), especially in the case of ‘Sencis’. Fe_3_O_4_ NPs increased the Chl *a* and *b* fluorescence levels to more than 0.04 to 0.3 × 10^6^ compared to control samples. ‘Quench’ seedlings had increased levels (0.61 to 0.63 × 10^6^) depending on the nanoparticle concentration. The 20 mg/L NP dose was optimal for increasing Chl *a* and *b* fluorescence to 0.84 and 0.42 × 10^6^, respectively, in treated ‘Abava’ plants in comparison to the control (0.77 and 0.32 × 10^6^, respectively). The content of Chl *a* and *b* in comparison to the control was higher after application of 10 and 20 mg/L Fe_3_O_4_ NPs (Figure 4C,D). Total Chl concentration increased in all treated cultivars (Figure 4E). The highest increasing of total Chl concentration was observed in ‘Quench’ seedlings, above 12.22 to 32.1 mg/g in comparison to control. However, the lowest increasing was observed in ‘Abava’ seedlings, above 2.49 to 9.91 mg/g compared to control samples. Moreover, it was observed that Chl content was inversely related to the Fe_3_O_4_ NPs concentrations, there was an observed decrease in total Chl concentration (in three different barley cultivars) with increasing NP concentrations from 1 to 20 mg/L. Previously, significant mean Chl fluorescence increases were detected in yellow medick seedlings treated with Fe_3_O_4_ NPs at 1, 2, and 4 mg/L [12].

Chloroplasts are very sensitive to iron oxide NPs [61]. Fe NPs may enhance the photosynthetic efficiency of crop plants [50,63]. Moreover, Fe ions released from iron oxide NPs may be utilised as a nutrition source in plants [61]. According to Yuan et al. (2018) [50], Fe NPs at lower concentrations significantly increased chloroplast number per mesophyll cell. Moreover, experiments showed that chloroplast ultrastructure could be changed by different concentrations of Fe [50]. The same researchers suggested that increased Chl content may promote photosynthesis activities in plant cells, which was also demonstrated in our study; Chl quality increased in barley seedlings after treatment with Fe_3_O_4_ NPs. Pariona et al. (2017) [52] indicated that Chl content in oak plants treated with Fe_3_O_4_ NPs (up to 160 nm) for 12 weeks had been significantly increased (up to 29.8%). This suggests that Fe_3_O_4_ NPs provide iron, which is involved in increasing Chl concentration [52]. Ghafariyan et al. (2013) [60] suggested that the biosynthesis of Chl *a* and *b* is influenced differently by iron oxide NPs. The presence of Fe_3_O_4_ NPs (80–100 nm) at a very low concentration (10^−3^ mg/L) enhanced Chl *a* and *b* content, biomass, grain yield, and activity of antioxidant enzymes in barley plants [64]. Li et al. (2021) [65] found that foliar spraying with appropriate concentrations of Fe_3_O_4_ NPs (20–50 mg/L) effectively enhanced the Chl content in medical plants. Furthermore, Trujillo-Reues et al. (2014) [59] did not detect differences in Chl content in lettuce exposed to Fe_3_O_4_ NPs (50–60 nm) at 10 and 20 mg/L for 15 days. In contrast, Tombuloglu et al. (2020) [13] obtained the opposite results. Interestingly, barley seedlings treated with α-Fe_2_O_3_ NPs (average size 14 nm) for three weeks showed a significant decrease in Chl content compared to the control, suggesting α-Fe_2_O_3_ NP phytotoxicity in barley [13]. Additionally, Fe_3_O_4_ NPs (20 nm) at 50 mg/L significantly decreased the Chl content in pummelo seedlings after 20 days of exposure [61]. The same results were obtained by Martínez-Fernández et al. (2016) [66], where Chl content was lower in sunflower seedlings grown hydroponically and exposed to Fe_2_O_3_ NPs for 5 days compared to the control. However, stress was not detected in treated sunflower plants. The authors explained this by the possible reduction of root hydraulic conductivity and thereby nutrient uptake [66]. Our results of total Chl content can also be explained with the above-mentioned reason. Differences in application position, growth environment, and plant species may explain these differences in the impact of Fe_3_O_4_ NPs on Chl content [65].

The assessment of Chl content and fluorescence parameters are needed to estimate the photosynthetic rates of CO_2_ assimilation under stress and determine plants’ response or tolerance to environmental stress [61,65,67,68]. Moreover, Yang et al. (2017) [69] and Li et al. (2021) [65] demonstrated significant linear correlations between Chl fluorescence/content and photosynthesis, indicating that increased Chl in plants subsequently increased plant photosynthetic efficiency [13,65]. Our results suggest that Fe_3_O_4_ NPs at low concentrations could be successfully used as nanonutrition for increasing barley photosynthetic efficiency and possibly enhancing yield.

### 3.4. Effect of Fe_3_O_4_ Nanoparticles on Genotoxicity in Barley Seedlings

**Figure 5:**
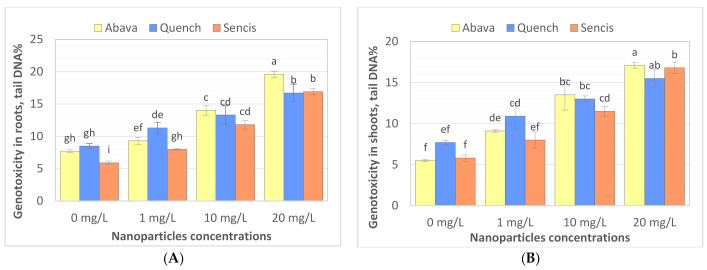
Evaluation the effect of Fe_3_O_4_ nanoparticles on barley genome DNA of three *H. vulgare* L. cultivars using comet assay for roots (**A**) and shoots. (**B**) Values are the mean of three replicates with SD. Different letters within each bar indicate significant differences at *p* < 0.05 and the same letters indicate no significant difference (Tukey’s test—two-way analysis of variance).

The comet assay is widely used to study the genotoxicity of various NPs in plants. Comet assay results are most often expressed as % tail DNA [70,71]. To the best of our knowledge, there are a limited number of studies performed on genotoxicity evaluation of Fe_3_O_4_ NPs in plants. Compared to the control, Fe_3_O_4_ NP treatments (1, 10, and 20 mg/L) significantly increased DNA damage (Figure 5A,B) in roots and shoots, particularly after treatment with a higher concentration of nanoparticles. In general, higher genotoxicity was observed in roots of both treated and control seedlings. Genotoxicity level increased with increasing of Fe_3_O_4_ NP concentrations. ‘Quench’ seedlings were the least sensitive to changes in DNA compared to the other two cultivars, there was observed about 7.8% higher genotoxicity at 20 mg/L compared with control samples. Genotoxicity level increased above 11.6% and 11.9% in ‘Abava’ seedlings’ shoots and roots, respectively, and above 11% in both ‘Sencis’ seedlings’ shoots and roots.

The same study indicated that Fe_3_O_4_ NP concentrations up to 70 mg/L did not show significant genotoxicity in barley seedlings treated with NPs for 2 weeks [53]. A study on garden rocket seedlings grown hydroponically with the addition of 1–4 mg/L of Fe_3_O_4_ NPs for 5 weeks presented an insignificant genome template stability decrease [58]. Furthermore, interesting genotoxicity results were obtained from yellow medick seedlings exposed to the same NPs hydroponically. Generally, genomic template stability significantly decreased in seedlings treated with NPs; however, there was an observed decrease in genotoxicity with increasing NP concentrations from 1 to 4 mg/L [12]. Moreover, our previous study on flax tissue cultures showed very low genotoxicity in callus cultures exposed to 0.5–1.5 mg/L Fe_3_O_4_ NPs [72]. Saquib et al. (2016) [70] indicated the genotoxicity of Fe_2_O_3_ NPs (22 nm) in radish. Dose-dependent DNA damage was observed with NP concentrations from 0.25 to 2 mg/mL.

Fe_3_O_4_ NPs in culture media take part in various oxidation-reduction reactions, which lead to Fe^3+^ and Fe^2+^ ion formation with the subsequent formation of different ROS (reactive oxygen species) [73,74]. ROS formation caused by the Fe NP oxidation process can also be destructive to plants [75]. However, oxidative stress from Fe deficiency is much stronger than that from Fe_3_O_4_ NPs [61]. During the NP sonication process, Fe ions are also released, however, Trujillo-Reues et al. (2014) [59] found that the concentration of Fe ions released from Fe_3_O_4_ NPs (at concentrations up to 20 mg/L) during sonication for 30 min is negligible, suggesting that the sonication process in our study (1–20 mg/L of NPs) did not affect the release of iron ions. It seems that the genotoxicity observed in this study could be related to Fe^3+^ and Fe^2+^ ion formation in the hydroponic medium before they were absorbed by barley seedlings.

### 3.5. Effect of Fe_3_O_4_ Nanoparticles on Barley miRNA Expression

**Figure 6:**
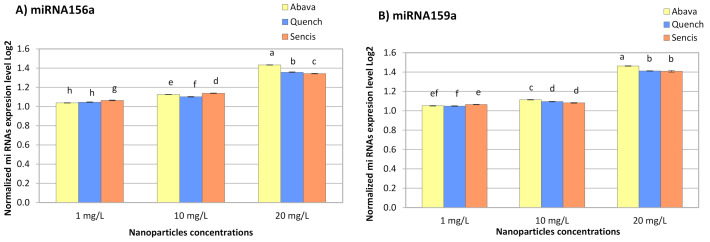
The results of the determination of miR156a (**A**), miR159a (**B**), expression levels in control and experimental groups of three *H. vulgare* L. cultivars (plants exposed to different concentrations of Fe_3_O_4_ nanoparticles). Values are the mean of three replicates with SD. Different letters within each bar indicate significant differences at *p* < 0.05 and the same letters indicate no significant difference (Tukey’s test—two-way analysis of variance).

The response of miRNA to metals depends on plant and metal species [76]. All concentrations of Fe_3_O_4_ NPs significantly increased miR156a and miR159a expression in the three barley varieties (Figure 6A,B). With an increase in NP concentration, the miRNA expression level also increased and ranged from 1.038- (Abava, 1 mg/L, miR156a) to 1.46-fold (Abava, 20 mg/L, miR159a).

In a Latvian barley variety treated with Fe_3_O_4_ NPs at 35 and 70 mg/L, a significant increase in miR156a expression was detected (1.96- and 3.75-fold, respectively) [53]. Additionally, there was a significant increase in miR159c expression in yellow medick after a 5-week exposure to small Fe_3_O_4_ NPs (1, 2, and 4 mg/L) [12]. The obtained results of another experiment with garden rocket also showed a slight increase in miR159c expression level [58]. The expression level of miR156 in pineapple plants exposed to MgO NPs at 1–4 g/mL was increased. However, miR159 expression was downregulated in the same plants [77]. Tabatabaee et al. (2021) [36] reported that Cu NPs at concentrations above 100 mg/L significantly increased the expression of miR159 in pepper seedlings.

According to a critical review by Yang and Chen (2013) [76], miR156 and miR159 were differently expressed in various plant responses to different heavy metal stressors. Thus, miR159 is downregulated in response to aluminium (Al), arsenic (As), mercury (Hg), cadmium (Cd), and manganese (Mn) in different plants. In turn, miR156 is upregulated by Al, As, and Mn and downregulated under Cd and Hg stress [76]. The same situation is relevant in plant responses to metal NPs. In switchgrass seedlings treated with 0.1–1% TiO_2_ NPs, the expression of miR156 and miR159 increased [78]. Yu et al. (2019) [79] indicated that miR156 and miR159 in wild barley seedlings might be related to Cd tolerance. According to Kantar et al. (2010) [28], hvu-miR156a expression in barley leaves was upregulated during dehydration stress. In *Arabidopsis*, miR156 targets are *squamosa promoter binding protein-like* (*SPL*) genes, which control plant development and physiology [80]. Moreover, Cui et al. (2014) [81] demonstrated that this miRNA in *Arabidopsis* takes part in mechanisms related to abiotic stress tolerance. Thus, miR156 overexpression increased drought stress tolerance. In turn, Križnik et al. (2020) [26] suggested that miR156 in *Nicotianas* can participate in plant resistance to viral infection. The targets of miR159 are *MYB* genes [82], which are transcription factor genes that control different processes, including responses to biotic and abiotic stress, as well as plant tolerance to metal stress [83,84]. Yao et al. (2021) [27] found that target genes of hvu-miR156a and hvu-miR159b in Tibetan hulless barley (*H*. *vulgare* variety *nudum* Hook. F.) are NAD(P)H-ubiquinone oxidoreductase B (NDB) and phosphatidylserine decarboxylase (PSD). miR156 and miR159 are fungi responsive miRNAs. Both miRNAs respond to wheat infection with powdery mildew [85]. Researchers found that infection with barley leaf stripe fungi affected the expression of miR156 and miR159 families. This means that these miRNAs can be used to improve resistance to barley leaf stripe [27]. Zhao et al. (2012) [82] reported that target genes of miR156 encoded plant disease resistance proteins, while targets of miR159 encoded peroxidase and cytokinin oxidase proteins. Several studies on miR156 and miR159 in plants showed that target genes of these miRNAs are involved in responses to various environmental stressors, such as fungal infection, cold, dehydration, drought, UV light, and mechanical stress [27,28,80,82,85,86]. miR156 and miR159 may also contribute to the interaction between barley and Fe_3_O_4_ NPs. Significant changes were observed between the three different barley genotypes. Therefore, a future study is necessary to explore the impact of Fe_3_O_4_ NPs on barley seedlings infected with *Blumeria graminis* to compare results obtained in this study with the same results from infected seedlings and to determine miR156, miR159, *mlo*, and *mla* gene expression in different genotypes of infected barley seedlings under Fe_3_O_4_ NPs stress.

## 4. Conclusions

Based on the data obtained in this study, 25 nm Fe_3_O_4_ NPs entered barley (*H*. *vulgare* L.) tissues. Fe_3_O_4_ NPs at concentrations of 1, 10, and 20 mg/L directly increased seedlings’ root length in three cultivars and reduced shoot lengths of ‘Quench’. The number of roots also was reduced after treatments. Total chlorophyll concentration was increased in all cultivars mostly in an inverse manner. Furthermore, the expression level of miRNA156a and miRNA159a in all three *H*. *vulgare* cultivars was increased. Moreover, these NPs increased the level of genotoxicity in the tested seedlings. It is important to note that each genotype could have an opposite reaction on the Fe_3_O_4_ NPs. These results are important to better understand the potential impact of Fe_3_O_4_ NPs at low concentrations in agricultural crops and the potential of these NPs as nanonutrition for enhancing barley growth and yield. The obtained results will be used in the future study of the effect of NPs on barley resistance-related and chlorophyll synthesis-related gene expression.

## Data Availability

The data presented in this study are available on request from the corresponding author.
